# A Study on the Relationship between Serum Beta 2-Microglobulin Levels, Underlying Chronic Kidney Disease, and Peripheral Arterial Disease in High-Vascular-Risk Patients

**Published:** 2012-12-15

**Authors:** Diego Real de Asúa, Ramón Puchades, Iluminada García-Polo, Carmen Suárez

**Affiliations:** 1 Vascular Risk Unit, Internal Medicine Department, Fundación de Investigación Biomédica, Hospital Universitario La Princesa, Madrid, Spain

**Keywords:** Ankle-Brachial Index, Beta 2-Microglobulin, Glomerular Filtration Rate, Peripheral Arterial Disease, Renal Insufficiency

## Abstract

**Background:**

Serum beta 2-microglobulin (B2M) levels have been found to be increased in patients with peripheral arterial disease (PAD), yet it is still unknown whether B2M correlates with PAD intensity.

**Objectives:**

We aim to evaluate the correlation between B2M and the ankle-brachial index (ABI) values in high-vascular-risk patients.

**Methods:**

This is a cross-sectional study of 63 high-vascular-risk patients admitted to the Cardiology Department or evaluated as outpatients in the Internal Medicine Department of our institution. Patients were classified into two groups according to their ABI: patients without PAD (n = 44, ABI values between 0.9 and 1.4) and patients with PAD (n = 19, ABI values lower than 0.9 or higher than 1.4). We performed univariate and multivariate analysis based on a multiple linear regression model.

**Results:**

Serum B2M levels were higher in patients with pathological ABI values than in those without PAD (2.36 ± 1.13 vs. 1.80 ± 0.65 mg/L; P<0.05). We found no correlation between B2M and ABI in our total population (r = –0.12) or in patients with PAD (r = –0.09; NS for both comparisons). Age, gender, arterial hypertension, estimated glomerular filtration rate (eGFR), uric acid, total cholesterol, and LDL-cholesterol correlated with B2M in the univariate analysis. In the final linear regression model, eGFR, uric acid and total cholesterol correlated independently with B2M (P<0.01).

**Conclusion:**

We found no correlation between B2M levels and ABI values in high-vascular-risk patients that could usefully help in the subsequent diagnosis of PAD. However, we observed a significant correlation between B2M and eGFR, even when renal function was only slightly impaired.

## 1. Background

Atherosclerosis is the main pathophysiological substrate of cardiovascular disease, the leading cause of morbidity and mortality in developed countries (1). Peripheral arterial disease (PAD) is one of the most common manifestations of atherosclerosis (2). Its prevalence increases with age and the presence of concomitant vascular risk factors, and it can affect as many as 25% of high-vascular-risk patients (3). Diagnosing PAD can prove challenging, since most patients are asymptomatic and the combination of a thorough clinical history and detailed physical examination is not sufficiently sensitive to identify asymptomatic patients (4-6).

The ankle-brachial index (ABI) is a simple and inexpensive tool for assessing vascular permeability in the lower limbs and has proven to be the most accurate, practical, and effective method to date for diagnosing PAD (7). However, it is limited by its inter-observer variability, which increases in conditions such as critical lower extremity ischemia (8). Moreover, its application is time-consuming, and primary care practitioners usually lack the specialized equipment and trained personnel to perform ABI measurements in the office setting.

Interest in the use of plasma proteins to better stratify vascular risk has been growing during the last decade. These molecules would not only help to manage patients with moderate vascular risk, but could also be useful prognostic markers (9,10). Beta 2-microglobulin (B2M) is a low-molecular-weight polypeptide of the major histocompatibility complex class I molecule on the surface of all nucleated cells, and can be found in body fluids under physiological conditions (11). Since the molecule is filtered at the glomerulus (95%) and avidly reabsorbed at the proximal tubule (99%), plasma B2M levels depend on their production rate and the patient’s glomerular filtration rate (GFR) (12-14). The rate of production of B2M can increase significantly in active neoplasms, infections, or autoimmune diseases (15, 16). Serum B2M levels are also elevated in patients with increased arterial stiffness and in patients with PAD due to several factors, such as declining renal function, repeated bouts of ischemia and reperfusion in the legs, and vascular inflammation (17,18).

It is still unclear whether B2M could be a useful marker for PAD and thus identify candidates to be evaluated by ABI (19). This strategy could increase the pretest positive likelihood ratio of the ABI and facilitate its implementation in the office setting. Early recognition of PAD could trigger intensive risk factor modification with different treatments (20). The present study aims to evaluate the correlation between serum B2M levels and the diagnosis of PAD according to ABI values in a cohort of high-vascular-risk patients.

## 2. Materials and Methods

### 2.1. Study design

This is a cross-sectional study on high-vascular-risk patients from two different populations recruited consecutively on a 1:1 ratio, which included patients admitted to the Cardiology Department, and patients evaluated at the Vascular Risk outpatient clinic of the Internal Medicine Department, both at Hospital Universitario La Princesa in Madrid, Spain. The recruitment period ran from January 2008 to June 2009. The study was conducted in accordance with the provisions of the Declaration of Helsinki and Good Clinical Practice guidelines. All patients provided their consent before any study procedure was performed.

### 2.2. Patients

Patients older than 40 years with grade 3 hypertension, three or more vascular risk factors, metabolic syndrome, diabetes mellitus or proven subclinical organ damage per review of their medical history were eligible for inclusion. All patients were recorded as having high vascular risk according to the directives of the European Society of Hypertension (21). Metabolic syndrome was defined according to the National Cholesterol Education Program (Adult Treatment Panel III)criteria (22). Patients with active cancer, with GFR lower than 30 mL/min, or with a previous kidney transplant, as well as those with HIV or any other acute infection, were excluded from the study, since these conditions are known to substantially modify B2M levels(12, 15, 16). GFR was estimated (eGFR) using the 4-variable Modification of Diet in Renal Disease equation (MDRD-4) (23).

The diagnosis of PAD was based on the numerical result of each patient’s ABI in accordance with the guidelines of the Inter-Society Consensus for the Management of Peripheral Arterial Disease (TASC II) (2). Further imaging tests were not performed to confirm the diagnosis of PAD. Patients were classified into two groups according to their ABI. This included patients without PAD, with ABI values between 0.9 and 1.4, and patients with PAD, with ABI values lower than 0.9 or higher than 1.4. Since patients with ABI higher than 1.4 also have an increased cardiovascular risk, which is considered equivalent to that of patients with ABI lower than 0.9, both were included in the same group for the final analysis (24-27).

### 2.3. ABI Measurement Technique

Before measurements were taken, patients were asked to lie for two to three minutes with their head raised to 45º. Blood pressure (BP) in the arms was measured using an automated oscillometric device (OMRON-M6, Omron Healthcare, Vernon Hills, Illinois, USA). Three consecutive measurements were taken in both arms separated by one to two minute intervals. Only the results from the arm with the highest BP were used for the ABI determination. BP in the legs was measured using an 8-MHz duplex device (Huntleigh-SD2, Huntleigh Healthcare Ltd., Cardiff, UK). Three consecutive measurements separated by one to two minute interval were taken at the level of the posterior tibial and dorsalis pedis arteries of both ankles. To determine BP in the lower limbs, the average of the second and third measurements were used at each location. The highest systolic BP in each ankle was selected as the numerator for the ABI of that limb. The first BP measurement in the arm with the highest BP was selected as the denominator for the ABI. All ABI determinations were performed by the same two professionals, with experience in more than 30 ABI determinations prior to the beginning of the present study.

### 2.4. Beta 2-Microglobulin Measurement

Blood samples were collected from study subjects after 10-hour overnight fast. Serum was obtained by centrifugation (1500 g for 15 min), transferred into coded plastic tubes, rapidly frozen and stored at -20 ºC until analysis. B2M was measured using ORG 5 BM immunoassay (Orgentec Diagnostica GmbH, Germany). The intra-assay and inter-assay coefficients of variation were less than 3.6% and 4.9%, respectively.

### 2.5. Statistical Analysis

All data were processed using SPSS (SPSS 20.0.0, IBM Corp., Armonk, New York, USA) and GraphPad Prism software (GraphPad Software, San Diego, California, USA). Qualitative results are presented as absolute frequencies (percentages), whereas quantitative results are presented as means ± standard deviations (SD). Non-parametric tests were used when appropriate. We performed univariate and multivariate analysis using multiple linear regression to identify variables that correlated independently with B2M. Values exceeding the mean ± 2SD in the correlation analysis were considered outliers and were excluded. Variables whose statistical significance was lower than 0.1 in the univariate analysis were included in the final multivariate analysis. For the remaining results, P values less than 0.05 were considered significant. All statistical tests were two-tailed.

## 3. Results

### 3.1. Baseline characteristics

The study population included 63 patients recruited between January 2008 and June 2009. Baseline characteristics, including coexisting conditions, are presented in Table 1. Hypertension and dyslipidemia were the most prevalent vascular risk factors in both groups, and the coronary arteries were the most frequently damaged vascular territory. Patients with PAD (19/63 patients, 30%) were older, mostly females, with lower body mass index (BMI), suffered predominantly from type 2 diabetes mellitus (DM2), and had had an event in more vascular territories than patients without PAD. All patients were receiving antihypertensive and lipid-lowering medication during the study period.

**Table 1:** Baseline Characteristics. Results Are Presented as Means ± SD or as n (%).

Baseline characteristics		Total	Without PAD	PAD	*P* value
		n = 63	n = 44	n = 19	
Age (years)		65 ± 10.2	63 ± 10	71 ± 8	<0.01
Male gender, No. (%)		47 (75%)	37 (86%)	10 (53%)	<0.05
Weight (kg)		81.1 ± 17.2	85.2 ± 19.2	74.5 ± 10	<0.05
BMI (kg/m^2^)^a^		29 ± 4.8	29.7 ± 5.2	27.1 ± 2.8	<0.05
Normal weight		11 (17%)	6 (14%)	5 (26%)	NS
Overweight		25 (40%)	17 (39%)	8 (42%)	NS
Obese		21 (33%)	19 (43%)	2 (11%)	<0.05
Systolic BP (mmHg)		131 ± 22	129 ± 22	134 ± 23	NS
Diastolic BP (mmHg)		73 ± 13	76 ± 13	65 ± 9	<0.05
Heart rate (bpm)		69 ± 12	70 ± 12	68 ± 11	NS
Smoking status	Non-smoker	18 (29%)	13 (30%)	5 (26%)	NS
	Previous smoker	10 (16%)	7 (16%)	3 (16%)	NS
	Active smoker	35 (56%)	24 (55%)	11 (58%)	NS
Family history		12 (19%)	10 (23%)	2 (11%)	NS
Arterial hypertension		55 (87%)	37 (84%)	18 (95%)	NS
Dyslipidemia		51 (81%)	36 (82%)	15 (79%)	NS
T2DM ^a^		29 (46%)	17 (39%)	12 (63%)	<0.05
Metabolic syndrome		38 (60%)	26 (59%)	12 (63%)	NS
Hyperuricemia		26 (41%)	19 (43%)	7 (37%)	NS
Ischemic coronary disease		40 (63%)	28 (64%)	12 (63%)	NS
Cerebrovascular disease		9 (14%)	3 (7%)	6 (32%)	<0.05
Multiple territories		19 (30%)	2 (5%)	16 (84%)	<0.01
ABI		1.05 ± 0.38	1.09 ± 0.11	0.71 ± 0.17	<0.01

Abbreviations: PAD, peripheral arterial disease; BMI, body mass index; BP, blood pressure; T2DM: type 2 diabetes mellitus; NS, Non-significant.

^a^Normal weight, BMI between 18 and 25 kg/m^2^; Overweight, BMI between 25 and 30 kg/m^2^; Obese, BMI over 30 kg/m^2^.

The baseline analytical values of the study population are presented in Table 2. According to to the National Kidney Foundation criteria 23 patients (36%) had eGFR values >90 mL/min, 22 (35%) had eGFR values between 60 and 90 mL/min, and 15 patients (24%) had stage 3 CKD (22). Patients with PAD had significantly lower eGFR (58 ± 17 vs. 85 ± 25 mL/min, dif. means –27 ± 38 mL/min, 95%CI [–16.4 to –37.6]; P<0.01) and higher B2M levels (2.36 ± 1.13 vs. 1.80 ± 0.65 mg/L, dif. means 0.55 ± 0.26 mg/L, 95%CI [0.01 - 1.10]; P<0.05) than patients without PAD.

**Table 2:** Baseline Analytical Values in the Study Population. Results Are Presented as Mean ± SD.

	Total	Without PAD	PAD	*P* value	Units
	n = 63	n = 44	n = 19		
Glucose	6.60 ± 2.16	6.44 ± 1.94	6.99 ± 2.66	NS	mmol/L
Total cholesterol	4.25 ± 1.24	4.40 ± 1.32	3.86 ± 0.96	NS	mmol/L
LDL-cholesterol	2.25 ± 1.01	2.36 ± 1.11	2.02 ± 0.73	NS	mmol/L
HDL-cholesterol	1.22 ± 0.34	1.22 ± 0.34	1.19 ± 0.34	NS	mmol/L
Triglycerides	1.60 ± 1.08	1.72 ± 1.24	1.34 ± 0.56	NS	mmol/L
Uric acid	368.8 ± 95.2	327.1 ± 83.3	368.8 ± 101.1	NS	mmol/L
Creatinine	88.4 ± 26.5	88.4 ± 26.5	97.24 ± 25.6	NS	mmol/L
eGFR	85 ± 34	85 ± 25	58 ± 17	<0.01	mL/min
B2M	1.97 ± 0.86	1.80 ± 0.65	2.36 ± 1.13	<0.05	mg/L

Abbreviations:eGFR, estimated glomerular filtration rate; B2M, beta 2-microglobulin; NS, non-significant

### 3.2. Correlation between Serum B2M Levels and ABI Values

**Figure 1:**
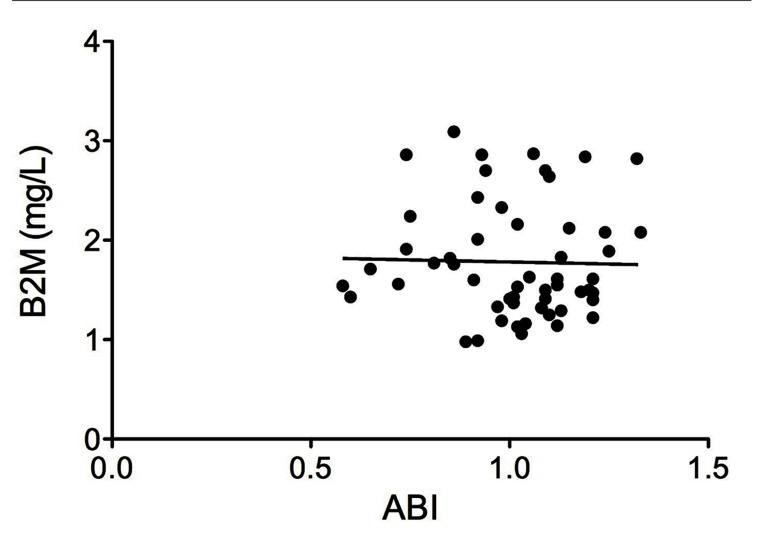
Correlation between Serum Beta 2-Microglobulin Levels and Ankle-Brachial Index in High-Vascular-Risk Patients B2M, beta 2-microglobulin; ABI, ankle-brachial index.

The correlation between B2M and ABI was assessed in 55 patients. Eight subjects with outlying values of B2M were excluded from the analysis. No significant correlation between B2M and ABI values was found in our total population (Figure 1, r = –0.12; not significant), nor in patients with PAD (r = –0.09; not significant).

**Figure 2:**
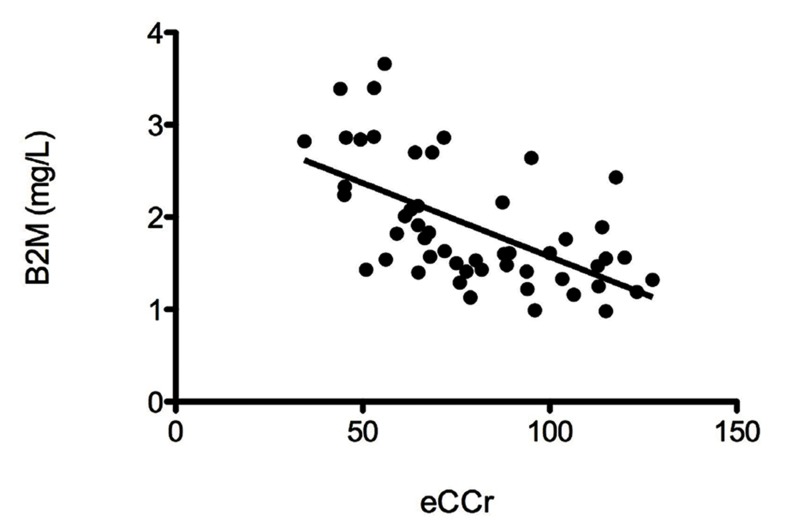
Correlation between Serum Beta 2-Microglobulin and Estimated Gomerular Filtration Rate in Patients with A High Vascular Risk B2M, beta 2-microglobulin; eCCr, estimated creatinine clearance.

In the univariate analysis, serum B2M levels correlated with age (r=0.49; P<0.01), femaleness (2.58 vs. 1.76 mg/L, dif. means 0.82 ± 0.31 mg/L, 95%CI [0.16 - 1.48]; P<0.05), arterial hypertension (2.05 vs. 1.44 mg/L, dif. means 0.61 ± 0.13 mg/L, 95%CI [0.35 - 0.87]; P<0.01), and plasma uric acid levels (r = 0.40; P<0.01). B2M showed an inverse strong correlation with eGFR (Figure 2, r = –0.62; P<0.01), and a fair one with total cholesterol (r = –0.31; P<0.05), and LDL-cholesterol (r = –0.27; P<0.05). Additionally, two variables approached a statistically significant correlation with B2M and were included in the final regression model, namely, family history of early vascular events (P=0.055) and type 2 diabetes mellitus (P=0.07). A statistically significant association was observed between gender and eGFR values, as the latter were significantly lower in women (64.7 vs. 92.3 mL/min, dif. means –27.6 ± 9.4 mL/min, 95%CI [–8.8 to –46.4]; P<0.01). No other relevant associations were observed.

The multivariate analysis is presented in Table 3. Serum B2M levels independently correlated with femaleness, uric acid, total cholesterol, and eGFR (ANOVA F 10.49; P<0.01). The adjusted R^2^ value for the model was 0.62.

**Table 3:** Independent Correlates of Serum Beta 2-Microglobulin in the Multivariate Analysis Using a Linear Regression Model

	Non-standardized coefficients		Standardized coefficients	t	*P* Value
	Beta	Standard error	Beta		
Constant	1.866	1.213		1.538	0.131
Age	0.009	0.012	0.103	0.746	0.460
Gender (male) ^a^	–0.726	0.206	–0.360	–3.526	<0.01
Family history	–0.339	0.191	–0.154	–1.778	0.082
Arterial hypertension	0.200	0.253	0.076	0.789	0.434
T2DM	0.251	0.161	0.140	1.557	0.127
eGFR ^a^	–0.007	0.004	–0.279	–2.040	<0.05
Uric acid ^a^	0.184	0.056	0.316	3.281	<0.01
Total cholesterol ^a^	–0.009	0.004	–0.490	–2.348	<0.05
LDL-cholesterol	0.009	0.004	0.393	1.934	0.060

Abbreviations: T2DM, type 2 diabetes mellitus; eGFR, estimated glomerular filtration rate.

^a^R^2^ for the model: 0.68. R^2^ (adjusted): 0.62.

## 4. Discussion

We found no correlation between B2M levels and ABI values in high-vascular-risk patients that could usefully help in the subsequent diagnosis of PAD. However, the results of this study show a significant correlation between serum B2M levels and eGFR, even when renal function is only slightly impaired.

To our knowledge, two other studies have specifically addressed this issue. Wilson et al. were the first to reveal a correlation between ABI and B2M (18). Although our main results disagree with their findings, their analysis of the correlation between B2M and ABI did not exclude outliers. Several other works have also disputed the initial findings of these authors. Our results agree with those of Kals et al., who found no association between B2M or pulse wave velocity and ABI in a population similar to that of our study (17). Busti et al. found no changes in B2M levels in PAD patients after exercise on a treadmill, thus challenging the initial hypothesis by Wilson et al. of an increase in B2M levels in patients with PAD due to repeated bouts of ischemia-reperfusion (28).

Estimated GFR values were independently correlated with B2M levels in the regression analysis of the above-mentioned studies (17,18). The relationship between serum B2M levels and GFR is well documented. End-stage chronic kidney disease (CKD) leads to accumulation of B2M in peripheral tissues, which is an established cause of systemic amyloidosis in patients undergoing dialysis (29). The present results prove that this relationship exists even in the normal range of GFR. Although over 70% of our patients had normal or mildly impaired eGFR, this variable still had the strongest correlation with B2M. Moreover, the significant influence of gender (female) on B2M levels observed in our study was also mediated by a lower eGFR. The relationship between eGFR and B2M has also been described in other clinical settings, such as HIV infection, diabetes mellitus, and glomerulonephritis (30-33).

There is increasing evidence of the role of hyperuricemia in vascular damage, not only in the pathogenesis of hypertension, but also in metabolic syndrome and CKD (34). We interpreted the correlation between B2M and uric acid as a new indirect sign of the relationship between hyperuricemia and vascular risk. Surprisingly, we did not find type 2 diabetes mellitus to be significantly associated with B2M in our final multivariate model. The high burden of concomitant vascular risk factors could have accounted for a lower proportional significance of diabetes as a risk factor for PAD in these patients. Furthermore, the fact that other well-known risk factors, such as hypertension or smoking, were not found to be more prevalent in patients with PAD than in those without this diagnosis also underlines the high overall vascular risk of the study subjects. This effect was also observed in the largest study to date, which considered B2M as a marker for stratifying mortality in an elderly population, where Shinkai et al., found no significant correlation between B2M levels and the presence of DM2 after multiple statistical corrections for possible confounders (19). Given these results, which point to a non-specific elevation of B2M in patients with a high vascular risk, we agree with other authors that B2M levels may not indicate the presence of PAD, but may reflect an increased burden of systemic atherosclerosis in a setting of underlying CKD (14,17).

Our study is supported by a number of strong points. All the patients had a high vascular risk, even though not all of them had presented a vascular event at the time of the study. Prompt diagnosis of exacerbating factors is most needed in high-risk patients. All ABI determinations were performed by the two same professionals, thus diminishing potential inter-observer variability. The limitations of our study are mainly technical. ABI was determined using a mixed technique (oscillometric automated device for the arms and a duplex device for the legs). Small differences in measurements between the devices may have altered the final numerical value of the ABI. We did not apply a gold standard for GFR determination, and the estimation of this parameter with the MDRD-4 equation is slightly inaccurate for GFR values over 60 mL/min (35). The National Kidney Foundation also encourages its use over plasma creatinine levels across the complete range of GFR values (23), and estimated GFR with the MDRD-4 equation has been used to assess GFR in a number of authoritative studies (36-38). The use of ABI as the sole diagnostic tool to assess PAD may have minimally underestimated the real prevalence of the disease in our high-vascular-risk setting. Finally, the exclusion of patients with an eGFR below 30 mL/min and those with outlying values of B2M could have partially induced a bias to the null. However, it is precisely in patients with end-stage CKD in whom the correlation between B2M and eGFR has already been best described (12-14). Thus, the possible additional influence of other vascular risk factors on that correlation would probably be proportionally less significant.

It is still unknown whether the use of B2M as a biomarker could add any additional information to the classic models of vascular risk stratification, which are based on simpler clinical and analytical parameters, especially in patients with moderate vascular risk. Other biomarkers face similar challenges (9). To sum up, we failed to find a correlation between B2M levels and ABI values in high-vascular-risk patients that could usefully help in the subsequent diagnosis of PAD. However, we show a significant correlation between serum B2M levels and eGFR throughout the complete range of renal function values down to a lower cut-off value of 30 mL/min.
